# Desenvolvimento de Modelos de Cardiopatia Congênita Impressos em 3D Usando Fluxo de Trabalho Viável e de Baixo Custo

**DOI:** 10.36660/abc.20260186

**Published:** 2026-06-23

**Authors:** Hinpetch Daungsupawong, Viroj Wiwanitkit

**Affiliations:** 1 Private Academic Consulant Vientiane Laos Private Academic Consulant, Vientiane - Laos; 2 Saveetha Institute of Medical and Technical Sciences Saveetha Dental College Chennai Índia Saveetha Institute of Medical and Technical Sciences (Deemed to be University) Saveetha Dental College, Chennai – Índia

**Keywords:** Impressão Tridimensional, Cardiologia

**Prezado Editor,**

A publicação intitulada "Desenvolvimento de Modelos de Cardiopatias Congênitas em Impressão 3D utilizando Metodologia de Baixo Custo – Uma Potencial Ferramenta para Aprimorar o Ensino da Cardiologia Pediátrica^1^" é aqui discutida. Este estudo apresenta um conceito interessante de utilização da tecnologia de impressão 3D para criar modelos de baixo custo de cardiopatias congênitas, uma abordagem potencial para apoiar o aprendizado anatômico e fisiopatológico. No entanto, uma limitação significativa do estudo reside na sua avaliação de resultados, que se baseia principalmente em opiniões subjetivas dos participantes. Foram utilizados apenas questionários de satisfação de médicos residentes, sem medidas quantitativas de desempenho de aprendizagem, como pontuações pré e pós-teste ou comparações com outros recursos didáticos, como imagens 2D ou modelos digitais.

Além disso, o tamanho da amostra de avaliadores não foi claramente especificado, o que pode limitar a confiabilidade das conclusões sobre a eficácia educacional. Ademais, a classificação de "100% de concordância" em diversas questões pode refletir viés de desejabilidade social.

Metodologicamente, existem limitações quanto à validade anatômica do modelo. O processo de modelagem utiliza dados de tomografia computadorizada combinados com dados de válvulas cardíacas obtidos por ecocardiografia, utilizando técnicas de mapeamento de altura, o que pode levar a erros devido à integração de dados de duas fontes de imagem com resoluções e ângulos de visão diferentes. Além disso, o uso de impressoras 3D de mesa e materiais de baixo custo pode afetar a resolução estrutural, particularmente para pequenas estruturas como folhetos valvares ou trabéculas. A ausência de indicadores quantitativos quanto à precisão dimensional ou comparação com os dados de imagem originais, juntamente com análises estatísticas ou testes de diferenças entre modelos ou grupos de aprendizes, limita a interpretação dos resultados apenas a níveis descritivos.

Uma reinterpretação dos resultados do estudo sugere que os modelos 3D de baixo custo são valiosos não apenas pelo seu realismo anatômico, mas também desempenham um papel crucial como ferramenta para aumentar o envolvimento na aprendizagem. Esse envolvimento pode ser um fator-chave na percepção dos alunos sobre a compreensão das estruturas espaciais. A criação de um conjunto abrangente de modelos que cubram múltiplos tipos de cardiopatias congênitas também pode facilitar a aprendizagem comparativa da morfologia, benéfica para o desenvolvimento do raciocínio clínico. No entanto, essa eficácia deve ser investigada mais a fundo por meio de estudos experimentais que comparem esses modelos a métodos de ensino padrão, como a visualização digital em 3D ou a realidade virtual.

Questões para discussão futura incluem: Os modelos 3D de baixo custo superam a mídia digital a longo prazo? Qual é a resolução mínima necessária para que os modelos mantenham seu valor educacional? Qual a eficácia do uso desses modelos na comunicação com pacientes ou familiares? Além disso, a criação de um banco de dados de modelos de cardiopatia congênita de código aberto poderia ajudar a reduzir as disparidades educacionais entre instituições? Essa será uma direção fundamental para o desenvolvimento futuro dessa tecnologia.
