# Supplementary material for: Desenvolvimento de Modelos de Cardiopatia Congênita Impressos em 3D Usando Fluxo de Trabalho Viável e de Baixo Custo
Source: Arq Bras Cardiol. 2026 Jun 23;123(5):e20260186. [Article in Portuguese] doi: 10.36660/abc.20260186 (PMC13399137; doi:10.36660/abc.20260186)
Supplement: Translation

# Translation: Development of 3D-Printed Congenital Heart Disease Models Using Feasible Low-Cost Workflow

**DOI:** 10.36660/abc.20260186i

**Authors:** Hinpetch Daungsupawong, Viroj Wiwanitkit

**Affiliations:** 1 Vientiane Laos Private Academic Consulant, Vientiane - Laos; 2 Chennai India Saveetha Institute of Medical and Technical Sciences (Deemed to be University) Saveetha Dental College, Chennai – India

**Keywords:** Three-Dimensional Printing, Cardiology

**Dear Editor**

The publication on "Development of 3D-Printed Congenital Heart Disease Models Using Feasible Low-Cost Workflow - A Potential Tool to Improve Pediatric Cardiology Education^1^" is hereby discussed. This study presents an interesting concept of using 3D printing technology to create low-cost models of congenital heart disease, a potential approach to support anatomical and pathophysiological learning. However, a significant limitation of the study lies in its evaluation of outcomes, which relies primarily on subjective opinions from participants. Only resident physician satisfaction questionnaires were used, without quantitative learning achievement measures such as pre- and post-test scores or comparisons with other teaching aids like 2D images or digital models. Furthermore, the sample size of the evaluators was not clearly specified, potentially limiting the reliability of conclusions regarding educational effectiveness. Additionally, the "100% agreement" rating on several issues may reflect social desirability bias.

Methodologically, there are limitations regarding the anatomical validity of the model. The modeling process uses data from CT scans combined with heart valve data from echocardiography using height mapping techniques, which may lead to errors due to the integration of data from two image sources with different resolutions and viewing angles. Moreover, the use of desktop 3D printers and low-cost materials may affect structural resolution, particularly for small structures such as valve leaflets or trabeculations. The lack of quantitative indicators regarding dimensional accuracy or comparison with original imaging data, along with statistical analysis or testing for differences between models or learner groups, limits the interpretation of the results to descriptive levels only.

A reinterpretation of the study results suggests that low-cost 3D models are valuable not only for their anatomical realism but also play a crucial role as a tool for enhancing learning engagement. This engagement may be a key factor in learners’ perceived understanding of spatial structures. Creating a comprehensive set of models covering multiple CHD types may also facilitate comparative morphology learning, beneficial for developing clinical thinking skills. However, this effectiveness should be further investigated through experimental study designs comparing these models to standard teaching methods, such as digital 3D visualization or virtual reality.

Future discussion questions include: Do low-cost 3D models outperform digital media in the long term? What is the minimum resolution required for models to maintain educational value? How effectively can the use of these models impact communication with patients or families? Furthermore, could the creation of an open-source CHD model database help reduce educational disparities between institutions? This will be a key direction for the future development of this technology.
