# Supplementary material for: Desenvolvimento de Modelos de Cardiopatia Congênita Impressos em 3D Usando Fluxo de Trabalho Viável e de Baixo Custo
Source: Arq Bras Cardiol. 2026 Jun 23;123(5):e20260186. [Article in Portuguese] doi: 10.36660/abc.20260186 (PMC13399137; doi:10.36660/abc.20260186)
Supplement: Related texts

# Reply: Carta-resposta

**Authors:** Francisco Candido Monteiro Cajueiro

**Affiliations:** 1 Universidade de Pernambuco Recife PE Brasil Universidade de Pernambuco, Recife, PE – Brasil

**Prezado Editor,**

Agradecemos ao autor pelo interesse em nosso trabalho e pelos comentários pertinentes sobre nosso estudo acerca do desenvolvimento de modelos de cardiopatia congênita (CC) impressos em três dimensões (3D) de baixo custo.

Nosso estudo foi concebido com um objetivo específico e claramente definido: descrever um fluxo de trabalho viável, reproduzível e de baixo custo para o desenvolvimento de modelos de cardiopatia congênita impressos em 3D e fornecer um recurso educacional compartilhável. Nesse contexto, o trabalho foi intencionalmente projetado como um estudo descritivo, no estilo "faça você mesmo", com foco na acessibilidade e na implementação prática, em vez de validação quantitativa ou análise comparativa de desempenho. Acreditamos que essa distinção seja essencial para a interpretação adequada de nossos resultados.

O crescente interesse na impressão 3D em cardiologia levou ao desenvolvimento de modelos altamente sofisticados com excelente fidelidade anatômica, frequentemente utilizando softwares avançados, integração de imagens multimodais e materiais de alto custo. Embora essas abordagens representem avanços importantes, podem não ser facilmente acessíveis em muitas instituições, particularmente em países de baixa e média renda. Por esse motivo, estratégias de baixo custo têm sido cada vez mais exploradas, demonstrando que fluxos de trabalho simplificados, baseados em softwares de código aberto e impressão 3D de mesa, ainda podem produzir modelos com significativo valor educacional.

Nesse contexto, as escolhas metodológicas adotadas em nosso estudo (incluindo o uso de hardware de baixo custo, representações anatômicas simplificadas e técnicas alternativas para modelagem de válvulas) foram deliberadas e alinhadas com o propósito dos modelos. Conforme explicitamente declarado no manuscrito, esses modelos não foram projetados para tomada de decisões clínicas ou planejamento cirúrgico, mas sim como ferramentas educacionais destinadas a aprimorar a compreensão espacial, a comunicação e o engajamento entre alunos e o público não especializado.

A estratégia de avaliação também foi alinhada a esse objetivo. A avaliação qualitativa, realizada por médicos especialistas e residentes, foi utilizada para explorar a usabilidade, a clareza e os benefícios educacionais percebidos. Embora reconheçamos as limitações inerentes à avaliação subjetiva e o potencial de viés, esses aspectos foram considerados e discutidos no manuscrito. Em estágios iniciais de inovação educacional, essas abordagens fornecem informações valiosas sobre a experiência do usuário e a aplicabilidade no mundo real.

Reconhecemos também que estudos adicionais que incorporem validação quantitativa, delineamentos comparativos e avaliação a longo prazo da retenção de conhecimento fortaleceriam ainda mais a base de evidências nesta área. Essas direções representam importantes oportunidades para pesquisas futuras e estavam além do escopo do presente estudo, que se concentrou principalmente na viabilidade e acessibilidade.

Uma importante contribuição do nosso trabalho é o compartilhamento aberto dos modelos desenvolvidos por meio de uma plataforma de acesso livre (https://sites.google.com/view/3dpequenoscoracoes), permitindo a replicação, adaptação e disseminação em diferentes contextos. Acreditamos que essa abordagem pode ajudar a ampliar o acesso a ferramentas educacionais em cardiologia congênita, principalmente em locais com recursos limitados, além de contribuir para a democratização mais ampla de tecnologias educacionais avançadas em medicina cardiovascular.

Além disso, gostaríamos de destacar um breve editorial recente que aborda o papel da impressão 3D de baixo custo na educação sobre cardiopatias congênitas, o qual oferece uma perspectiva ponderada e equilibrada sobre várias das questões aqui discutidas.^1^ Também recomendamos aos leitores interessados uma entrevista em vídeo relacionada, que explora mais a fundo as motivações e os aspectos práticos dessa iniciativa.^2^

Em resumo, nosso estudo visa complementar os avanços existentes na impressão 3D, demonstrando que recursos educacionais relevantes podem ser desenvolvidos utilizando métodos acessíveis. Agradecemos a oportunidade de esclarecer esses pontos e de contribuir para o debate em curso sobre estratégias inovadoras e inclusivas na educação sobre cardiopatias congênitas.

## References

[1] Ramos V (2026). The Role of Low-Cost 3D Printing for Education in Congenital Heart Diseases. Arq Bras Cardiol.

[2] ABC Cardiol (2026). ABC Spotlight – Dr. Francisco Cajueiro [Internet].

# Reply

**Authors:** Francisco Candido Monteiro Cajueiro

**Affiliations:** 1 Recife PE Brazil Universidade de Pernambuco, Recife, PE – Brazil

**Dear Editor,**

We thank the author for their interest in our work and for the thoughtful comments regarding our study on the development of low-cost three-dimensional (3D) printed congenital heart disease (CHD) models.

Our study was conceived with a specific and clearly defined objective: to describe a feasible, reproducible, and low-cost workflow for the development of 3D-printed CHD models and to provide a shareable educational resource. In this context, the work was intentionally designed as a descriptive, "do-it-yourself"–style study, focused on accessibility and practical implementation rather than on quantitative validation or comparative performance analysis. We believe this distinction is essential for the appropriate interpretation of our findings.

The growing interest in 3D printing in cardiology has led to the development of highly sophisticated models with excellent anatomical fidelity, often relying on advanced software, multimodal imaging integration, and high-cost materials. While these approaches represent important advances, they may not be readily accessible in many institutions, particularly in low- and middle-income settings. For this reason, cost-effective strategies have been increasingly explored, demonstrating that simplified workflows based on open-source software and desktop 3D printing can still produce models with meaningful educational value.

Within this framework, the methodological choices adopted in our study (including the use of low-cost hardware, simplified anatomical representations, and alternative techniques for valve modeling) were deliberate and aligned with the intended purpose of the models. As explicitly stated in the manuscript, these models were not designed for clinical decision-making or surgical planning, but rather as educational tools aimed at improving spatial understanding, communication, and engagement among learners and non-specialist audiences.

The evaluation strategy was similarly aligned with this objective. Qualitative assessment by both expert physicians and trainees was used to explore usability, clarity, and perceived educational benefits. While we acknowledge the inherent limitations of subjective evaluation and the potential for bias, these aspects were recognized and discussed in the manuscript. In early-stage educational innovation, such approaches provide valuable insight into user experience and real-world applicability.

We also recognize that additional studies incorporating quantitative validation, comparative designs, and long-term assessment of knowledge retention would further strengthen the evidence base in this field. These directions represent important opportunities for future research and were beyond the scope of the present study, which was primarily focused on feasibility and accessibility.

An important contribution of our work is the open sharing of the developed models through a freely accessible platform (https://sites.google.com/view/3dpequenoscoracoes), allowing replication, adaptation, and dissemination across different contexts. We believe that this approach may help expand access to educational tools in congenital cardiology, particularly in resource-limited settings, while contributing to the broader democratization of advanced educational technologies in cardiovascular medicine.

In addition, we would like to highlight a recent short editorial addressing the role of low-cost 3D printing in CHD education, which provides a thoughtful and balanced perspective on several of the issues discussed here.^1^ We also refer interested readers to a related videocast interview that further explores the motivations and practical aspects of this initiative.^2^

In summary, our study aims to complement existing advances in 3D printing by demonstrating that meaningful educational resources can be developed using accessible methods. We appreciate the opportunity to clarify these points and to contribute to the ongoing discussion on innovative and inclusive strategies in congenital heart disease education.
